# Proteomic analysis of *post mortem* brain tissue from autism patients: evidence for opposite changes in prefrontal cortex and cerebellum in synaptic connectivity-related proteins

**DOI:** 10.1186/2040-2392-5-41

**Published:** 2014-07-30

**Authors:** Jantine AC Broek, Paul C Guest, Hassan Rahmoune, Sabine Bahn

**Affiliations:** 1 Department of Chemical Engineering and Biotechnology, University of Cambridge, Tennis Court Road, CB2 1QT Cambridge, UK; 2 Department of Neuroscience, Erasmus Medical Centre, Dr. Molenwaterplein 50, 3015 GE Rotterdam, The Netherlands

**Keywords:** Autism, Synaptic regulation, Molecular profiling, *Post mortem* brain, Proteome, Selected reaction monitoring mass spectrometry

## Abstract

**Background:**

Autism is a neurodevelopmental disorder characterized by impaired language, communication and social skills. Although genetic studies have been carried out in this field, none of the genes identified have led to an explanation of the underlying causes. Here, we have investigated molecular alterations by proteomic profiling of *post mortem* brain samples from autism patients and controls. The analysis focussed on prefrontal cortex and cerebellum as previous studies have found that these two brain regions are structurally and functionally connected, and they have been implicated in autism.

**Methods:**

*Post mortem* prefrontal cortex and cerebellum samples from autism patients and matched controls were analysed using selected reaction monitoring mass spectrometry (SRM-MS). The main objective was to identify significantly altered proteins and biological pathways and to compare these across these two brain regions.

**Results:**

Targeted SRM-MS resulted in identification of altered levels of proteins related to myelination, synaptic vesicle regulation and energy metabolism. This showed decreased levels of the immature astrocyte marker vimentin in both brain regions, suggesting a decrease in astrocyte precursor cells. Also, decreased levels of proteins associated with myelination and increased synaptic and energy-related proteins were found in the prefrontal cortex, indicative of increased synaptic connectivity. Finally, opposite directional changes were found for myelination and synaptic proteins in the cerebellum.

**Conclusion:**

These findings suggest altered structural and/or functional connectivity in the prefrontal cortex and cerebellum in autism patients, as shown by opposite effects on proteins involved in myelination and synaptic function. Further investigation of these findings could help to increase our understanding of the mechanisms underlying autism relating to brain connectivity, with the ultimate aim of facilitating novel therapeutic approaches.

## Background

Autism spectrum disorders (ASDs) are neurodevelopmental conditions characterized by impairments or alterations in socialisation, with language changes and restricted and/or repetitive behaviours. Recent studies have estimated the prevalence of autism at 1 in 110 with evidence for a strong gender bias. Approximately four times as many males as females are diagnosed with autism based on *Diagnostic and Statistical Manual of Mental Disorders* (DSM)-5 criteria. Children with autism commonly display abnormal development before the age of three years, particularly those with the regressive autism sub-type [1-3]. Despite the distressing effects that autism can have on the lives of patients and their families, the molecular basis of this condition remains largely unknown. Consequently, there are still no effective pharmacological interventions that can ameliorate the core symptoms of autism.

A number of previous studies have attempted to elucidate pathomechanisms associated with autism using imaging, genetic and transcriptomic approaches. Although results have been sparse and sometimes conflicting across studies, a consensus has emerged which suggests changes in brain connectivity and synaptic function in autism patients [1,4]. Specific genes which have been implicated include *NLGN1* (neuroligin) and *NRXN1* (neurexin) [4], and this has suggested that changes in local and distal connectivity may result from an imbalance of neuronal excitation and inhibition [5,6]. This may also involve dysfunction of myelination pathways, as shown by the finding of circulating antibodies against myelin basic protein (MBP) and myelin-associated glycoprotein (MAG) in some autism patients [7,8].

Proteomic profiling studies have shown that brain-derived neurotrophic factor (BDNF) and glial fibrillary acidic protein (GFAP) are altered in autism patients. BDNF is a growth factor which may be related to the increased brain volumes seen in some young children with autism [9]. This is consistent with the findings of imaging studies of autism subjects, which have identified aberrant white matter growth patterns [10-12]. GFAP is an astrocytic marker and astrocytes are known to be involved in synaptic connectivity and inflammation [13]. Increased astrocyte activity has been observed in ASD patients [14,15] and changes in inflammatory pathways have been observed in the cerebral cortex, white matter and cerebellum [16]. Furthermore, circulating autoantibodies have been detected against GFAP and other proteins involved in neuronal and synaptic functions, including neurotrophic factors and neuronal-axonal filaments [17,18]. Finally, changes in mitochondrial and energy pathways have also been reported [19], although it has been hypothesized that these changes may be secondary to an as yet unidentified disease process [20]. Several independent studies have corroborated that creatine kinase (CK), an enzyme important for energy homeostasis, is one of the most robust chemical changes in autism and this is likely to parallel changes in synaptic remodelling [21].

In order to extend these studies and increase our understanding of the proteins and biological pathways affected in autism, we have carried out a targeted proteomic profiling study of *post mortem* brain samples from individuals with autism compared to controls, using selected reaction monitoring mass spectrometry (SRM-MS). SRM is an accurate, reproducible and quantitative technique to measure predetermined sets of proteins within the femto- to attomolar concentration range [22,23]. This method has advantages over Western blot analysis as multiple readings are taken of each analyte compared to only one for Western blot analysis [24]. Furthermore, the targeting of peptide sequences analyzed in the SRM method makes this a highly specific and quantitative analytical method whereas the Western blot approach relies on antibody reactivity and, therefore, may result in non-specificity due to potential antibody cross reactivity [25]. Our main objective was to identify changes in protein expression levels and to explore whether the affected proteins could be associated with the dysconnectivity hypothesis of autism.

## Methods

### Subjects

*Post mortem* prefrontal cortex (PFC) (Brodmann area 10; autism n = 10, controls n = 10) and cerebellum (CB) (lateral posterior and anterior lobe, autism n = 16, controls n = 17) were provided by the National Institute of Child Health and Human Development (NICHD) Brain and Tissue Bank for Developmental Disorders (University of Maryland School of Medicine, Baltimore, MD, USA). Patients were matched to controls with respect to age of death (PFC: *P* = 0.99; CB: *P* = 0.80), gender (PFC: *P* = 1.00; CB: *P* = 0.70), *post mortem* interval (PMI) (PFC: *P* = 0.08; CB: *P* = 0.18) and brain pH (PFC: *P* = 0.51; CB: *P* = 0.66), which is indicated in the additional files (see Additional file 1 for PFC and Additional file 2 for CB). The analysis of prefrontal cortex included samples from 10 autism individuals (age = 21.3 ± 4.3 years, PMI = 22.1 ± 3.9 hours) and 10 matched controls (age = 21.4 ± 4.3 years, PMI = 14.7 ± 0.8 hours), and cerebellum tissue included 14 individuals with a diagnosis of autism (age = 18.6 ± 3.4 years, PMI = 22.7 ± 3.5 hours) and 17 matched controls (age =17.5 ± 2.9 years, PMI = 16.7 ± 1.6 hours) (Table 1). The study was approved by the Columbia University Medical School Institutional review board, consent was obtained from next of kin and all samples were de-identified and personal information anonymised. Diagnosis of autism was confirmed by the structured Autism Diagnostic Interview-Revised (ADI-R) carried out with the parents, the childhood autism rating scale (CARS), family history documentation, gene analysis to exclude genetic disorders and mutations, and neurological examination (Table 1). Subjects with a diagnosis of autism were included in the study. Local ethical approval for use of this tissue was granted by the Cambridgeshire Local Research Ethics Committee.

**Table 1:** Demographic data for control and autism subjects

**Diagnosis**	**Gender**	**Age**	**PMI (hours)**	**Ethnicity**	**Medication history**	**Brain area**
control	male	12	16	Caucasian	NA	CB
control	male	20	18	Caucasian	NA	PFC
control	male	37	12	African American	marijuana	PFC, CB
control	male	9	36	Caucasian	NA	CB
control	female	19	29	Caucasian	none	CB
control	female	20	9	Caucasian	none	CB
control	male	46	13	Caucasian	NA	PFC, CB
control	male	5	18	African American	NA	CB
control	male	8	16	African American	NA	PFC, CB
control	male	20	19	Caucasian	NA	CB
control	female	4	15	African American	NA	CB
control	male	22	13	African American	none	PFC, CB
control	female	16	13	Caucasian	NA	PFC, CB
control	male	33	16	Caucasian	NA	PFC, CB
control	male	15	12	Caucasian	NA	CB
control	female	16	11	Caucasian	NA	PFC, CB
control	male	13	19	Caucasian	NA	PFC, CB
control	male	3	16	Hispanic	none	PFC, CB
autism	female	20	50	Caucasian	NA	CB
autism, MR	male	9	12	African American	Zyprexa, Reminyl	CB
autism, S	male	11	27	Hispanic	NA	PFC, CB
autism	female	4	13	African American	NA	CB
autism	male	9	16	African American	NA	CB
autism^a^	male	7	20	African American	NA	CB
autism	male	14	9	Caucasian	NA	CB
autism, MR	male	20	14	Caucasian	Naltrexone	PFC, CB
autism	male	38	26	African American	Respirdal, Luvox	PFC, CB
autism, S	male	46	29	Caucasian	NA	PFC, CB
autism	male	7	3	Caucasian	NA	PFC
autism	male	22	18	African American	Risperdal	PFC, CB
autism, S	female	16	13	Caucasian	Depakote, Keppra, Vitamin B6 and Prozac	PFC, CB
autism, MR	male	33	50	Caucasian	Seroquel, Prozac, Depakote, Geodon	PFC, CB
autism, S	male	16	20	Caucasian	Risperdal, Luvox, Clonidine and Insulin	PFC, CB
autism	male	4	21	Caucasian	NA	PFC, CB

ID = identification code, PFC = prefrontal cortex, CB = cerebellum, MR = mental retardation, S = seizure, NA = Not Applicable for control subjects and Not Available for cases. ^a^This sample was removed as an outlier and not considered in further statistical analyses.

### Sample preparation

All biochemicals and reagents were obtained from Sigma-Aldrich (Poole, UK) unless specified otherwise. Brain tissues (approximately 30 mg) were sectioned using a Leica Cryostat (Milton Keynes, UK), collected into pre-chilled lysing matrix D tubes (MP Biomedicals; Cambridge, UK) and stored at -80°C until use. Protein extraction was performed by addition of fractionation buffer (7 M urea, 2 M thiourea, 4% 3-[(3-cholamidopropyl)dimethylammonio]-1-propanesulfonate, 2% ASB14 and 70 mM dithiotreitol (DTT), followed by sonication for 10 seconds using a Branson Sonifier 150 (Thistle Scientific; Glasgow, UK) and vortexing for 30 minutes at 4°C. The homogenates were centrifuged for 3 minutes at 17,000 *g* and the supernatants collected for precipitation of the proteins using 4:1 volumes ice-cold acetone. The resulting pellets were suspended in 100 μL of 50 mM NH_4_HCO_3_ (pH 8.0). Sulfhydryl groups on proteins were reduced by incubation with 100 mM DTT for 30 minutes at 60°C and alkylated with 200 mM iodacetamide for 30 minutes at 37°C. Proteins were cleaved into peptides by incubation with 1:50 (trypsin:protein) porcine trypsin (Promega; Madison, WI, USA) for 17 hours at 37°C and stopped after 16 hours by addition of 0.80 μL of 8.8 M HCl. Samples were stored at -80°C. Prior to mass spectrometry analyses, 0.1% formic acid was added to a final concentration of 0.12 μg/μL protein.

### SRM-MS

Digested prefrontal cortex and cerebellum proteomes were analysed using SRM-MS on a Xevo TQ-S mass spectrometer coupled to a nanoAcquity UPLC system (Waters Corporation, Wilmslow, UK), as described previously [23]. Protein candidates were selected for this analysis given their association with pathways which have been implicated previously in autism. Physiochemical criteria for selecting tryptic peptides were based on peptide count, uniqueness and quality of transitions. All SRM functions had a 6 minute window of the predicted retention time and scan times were 0.02 seconds. Two peptides were selected for each target protein and isotopically-labelled peptides were synthesized at JPT Peptide Technologies GmbH (Berlin, Germany). The samples and labelled peptides were mixed together and separated using the following 42 minutes gradient: 97/3% (A/B) to 70/30% in 20 minutes; 70/30% to 15/85% in 5 minutes; and in 3 minutes to 97/3%. Peptide spectra were acquired in SRM mode using a capillary voltage of 2.35 kV and a cone voltage of 33 V. At least three transitions were measured for each peptide. The candidate proteins were identified with the SRM method as described previously [23].

### Statistical analyses

Statistical analyses of SRM data were conducted in the R statistical programming language (version 2.15.3; http://www.r-project.org), using the package MSstats [26-28]. Data processing consisted of log_2_ transformation to stabilize the variance and normalize peak intensities. The data were then analysed using a linear mixed model to detect proteins or peptides that were present at significantly different levels in cases compared to controls (autism/control). In the model, the experimental characteristics were linked to the data in terms of biological condition, labelling state, technical and biological replication and potentially interfering transitions [28]. The scope of inference for both biological replicates and technical replicates were set to ‘restricted’, as conclusions about the data are confined to the study itself. Furthermore, additional model interactions were performed for interference transitions. These analyses resulted in *P*-values, *Q*-values (allowing for multiple testing) and ratio changes (autism/control).

## Results

### Sample quality

SRM-MS analysis of prefrontal cortex protein extracts from autism and control patients showed no batch effects as indicated by principal component analysis (PCA), of which an additional plot shows this in more detail (Additional file 3). Conversely, analysis of the cerebellum protein extracts led to the identification of one outlying sample. After removal of this sample, no further outliers were detected, as is visualised in the additional PCA plot (Additional file 4). All further statistical analyses were carried out using only the remaining samples.

### SRM-MS detection

SRM-MS was used for targeted analysis of proteins associated with energy metabolism, astrocyte function, myelination and synaptic vesicles in accordance with literature evidence [4-21]. The proteins which were detectable using SRM-MS analysis are indicated in Table 2. GFAP, vimentin (VIME), creatine kinase B-type (CKB), MAG, MBP, myelin-oligodendrocyte glycoprotein (MOG), myelin proteolipid protein (PLP1), dynamin-2 (DNM2), syntaxin-1A (STX1A), syntaxin-binding protein 1 (STXBP1), synapsin-2 (SYN2), synaptotagmin-1 (SYT1), protein kinase C casein kinase substrate 1 (PACSIN1) were detected in both prefrontal cortex and cerebellum from all patients and controls. However, reelin (RLN) was only identified in the cerebellum and was therefore not used in the comparative analysis.

**Table 2:** **SRM-MS testing of targeted candidate proteins in *post mortem *** **prefrontal cortex and cerebellum from autism patients compared to matched controls**

			**Prefrontal cortex**	**Cerebellum**				**Prefrontal cortex**
**Pathways**	**Gene**	**Protein name**	**ratio**	***P*-value**	**Pathways**	**Gene**	**Protein name**	**ratio**
Cell adhesion	*RELN*	Reelin	ND	ND	Cell adhesion	*RELN*	Reelin	ND
Astrocyte	*GFAP*	Glial fibrillary acidic protein	1.02	0.7399	0.7968	1.14	1.8E-05^a^	7.42E-5
	*VIME*	Vimentin	0.69	2.8E-5^a^	0.0002	0.56	1 < E-10^a^	1 < E-9
Energy	*CKB*	Creatine kinase B-type	1.73	1.3E-9^a^	1.82E-8	0.92	0.2605	0.3777
Myelin	*MAG*	Myelin-associated glycoprotein	0.76	0.0087^a^	0.0272	1.30	0.0016^a^	0.0042
	*MBP*	Myelin basic protein	0.85	0.1440	0.2373	1.41	0.0003^a^	0.0009
	*MOG*	Myelin-oligodendrocyte glycoprotein	0.81	0.0567	0.1222	1.37	0.0002^a^	0.0006
	*PLP1*	Myelin proteolipid protein	0.82	0.0143^a^	0.0400	1.37	2.8E-10^a^	2.69E-9
Synaptic vesicle	*DNM2*	Dynamin-2	1.05	0.7811	0.8024	0.87	0.2910	0.4018
	*STX1A*	Syntaxin-1A	1.07	0.2086	0.3165	0.88	0.0102^a^	0.0227
	*STXBP1*	Syntaxin-binding protein 1	1.28	0.1002	0.1753	0.97	0.8031	0.8626
	*SYN2*	Synapsin-2	1.29	0.0486^a^	0.1135	1.04	0.6499	0.7539
	*SYT1*	Synaptotagmin-1	1.06	0.4092	0.5456	0.87	0.0165^a^	0.0342
	*PACSIN1*	Protein kinase C casein kinase substrate 1	0.96	0.4540	0.5778	0.84	8.1E-5^a^	0.0003

ND = not detected; ^a^significant *P*-value.

Indicated are the themes, protein name, the ratio (autism/control), the *P*-value and the *Q*-value. Grey shading indicates the proteins with a significant *P*-value or *Q*-value and a ratio of >10%.

### Comparative SRM-MS analysis of proteins in prefrontal cortex and cerebellum

**Figure 1:**
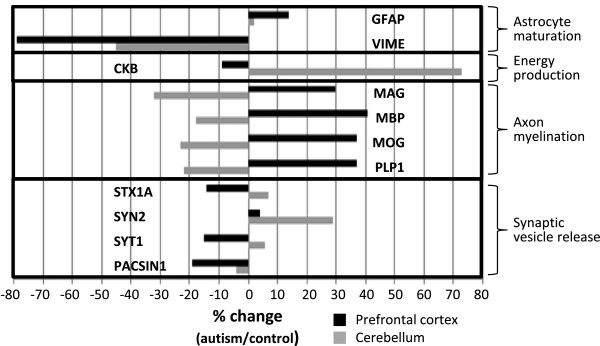
**Histogram showing selected reaction monitoring mass spectrometry (SRM-MS) analysis of proteins involved in astrocyte maturation, energy production, axon myelination and synaptic vesicle release.** The percent changes of each protein are shown for *post mortem* prefrontal cortex (black) and cerebellum (grey) tissues from autism patients compared to matched controls. The protein codes are defined in Table 2.

Prefrontal cortex and cerebellum showed a similar decrease in the levels of the immature astrocyte marker VIME, whereas the mature astrocyte marker GFAP was increased and this occurred only in the cerebellum (Figure 1). The energy-related CKB was increased by more than 70% in the prefrontal cortex and showed a non-significant decrease (*P* = 0.2605) in the cerebellum. The myelin-related proteins MAG and PLP1 were decreased in the prefrontal cortex with a concomitant increase in the synaptic proteins SYN2 and STBP1, although the latter was not significant (*P* = 0.1002). Conversely, the myelin related proteins MAG, MBP, MOG and PLP1 were all increased by more than 30% and the synaptic proteins STX1, SYT1 and PACSIN1 were decreased in the cerebellum. DNM2 was also decreased in the cerebellum although this was not significant (*P* = 0.2910).

## Discussion

This is the first report presenting results from a proteomics mass spectrometry study of rare *post mortem* brain tissues from autism patients and controls. Prefrontal cortex and cerebellum proteomes were investigated because a number of studies have already shown that these brain regions are affected in autism [29-32]. It has previously been suggested that impaired prefrontal cortex-cerebellar circuitry may be linked to autism symptoms [33]. In addition to its well known role in regulation of motor functions, it is now established that the cerebellum is also involved in regulation of cognition and other higher brain functions. Structural studies in non-human primates have shown that the cerebellum receives inputs through afferent nerves from several brain areas such as the prefrontal cortex which are known for their role in cognition and mood regulation [34-36]. Likewise, efferent nerves from the cerebellum have been traced to both motor and non-motor areas of the frontal cortex [37,38], which are routed through thalamic nuclei and complete the circuit [38]. Furthermore, functional evidence for a role of the cerebellum in higher brain function has been demonstrated by magnetic resonance imaging (MRI), which showed that activity in the cerebellar dentate nucleus correlated with changes in activity in the limbic system, parietal lobes and prefrontal cortex [39].

The main findings of this study provide evidence that molecular processes are differentially dysregulated in different brain regions in autism, which could affect various higher functions such as cognition, working memory, mood and emotions [40-42]. Here, the SRM-MS results showed decreased levels of proteins associated with myelination and increased levels of synaptic proteins in the prefrontal cortex, with opposite directional changes of the same proteins in the cerebellum. This is consistent with our unpublished observations showing decreased levels of myelin proteins in *post mortem* prefrontal cortex tissue in other psychiatric disorders, such as schizophrenia, bipolar and major depressive disorder. Furthermore, dysregulation of synaptic proteins may reflect alterations in synaptic density and a comparison with published data confirms the alterations of STX1A, STXBP1 and SYN2 in autism at the mRNA level [43]. In addition, SRM-MS showed an approximate 70% increase in the levels of CKB in the prefrontal cortex with a small non-significant decrease of this protein in the cerebellum. CKB has been used as an indicator of functional activation in magnetic resonance spectroscopy studies of the brain [44]. Increased myelination has an important role in promoting and maintaining axon integrity by increasing axonal calibre and thereby preventing sprouting and synaptic plasticity [45]. Moreover, decreased myelin thickness has previously been associated with disconnection of long-distance pathways, neighbouring connectivity and disruption of pathways involved in emotions [46]. Therefore, the current findings could suggest differential regulation of local synaptic connectivity in the prefrontal cortex and the cerebellum of autism patients.

It is possible that changes in local connectivity impair the transfer of information across different brain regions, given that the higher brain functions mentioned above require co-activation of networked brain areas [31]. Activation is known to be coordinated based on inter-regional relaying of signals through the connecting white matter tracts [47] and previous studies in autism have found changes in connectivity and overgrowth of brain tissues [40], along with alterations of white matter [6,48]. However, studies have shown that the patterns of white matter aberrations tend to differ depending on brain area, age and research techniques [6,40,48]. Therefore it is interesting that the present findings identified a difference in myelination-related protein levels in the prefrontal cortex and cerebellum. As white matter is mainly comprised of glial cells and myelinated axons, the current changes in myelin-related proteins may be associated with the proposed disconnectivity in autism. Likewise, we also identified decreased levels of the immature glial cell marker VIME in both brain regions and increased levels of GFAP in the cerebellum. This may be indicative of a relative loss of astrocyte precursor cells in the cerebellum of autism patients.

Cerebellar damage can result in verbal and communication deficits, as well as a reduction of higher-order executive functions and other cognitive abilities such as language processing, visuospatial abilities and attention [49]. Dysfunction of these pathways could be due to loss of cerebellar Purkinje cells, which has been observed in *post mortem* brains from autism patients compared to controls. Interestingly, this does not appear to be related to seizure activity as patients both with and without co-morbid epilepsy showed Purkinje cell loss [50,51].

One limitation of the present investigation was the low statistical power for detection of proteomic changes. This resulted from the limited number of *post mortem* samples available and the relatively wide age ranges of the subjects. However, the low numbers could not be avoided due to the scarcity of such high quality samples in brain banks. Moreover, the number of samples from female subjects is low due to the lower female prevalence [52]. Also, the different age groups studied could result in a masking of some molecular changes since previous studies have shown age dependent changes in the levels of many serum proteins in children and adolescents with autism [53]. Due to the rarity of these samples, the subjects were matched only for gender and age but not for drug treatment. Therefore, we cannot rule out the possibility that some of the changes may be medication or drug effects. Therefore, the presented findings should be considered preliminary and further validation studies should be carried out using larger sample sets, once these become available. This will require increased bio-banking efforts to allow studies involving stratification of samples by age and gender.

## Conclusion

The findings from our proteomics study suggest brain region-specific changes in local connectivity in the prefrontal cortex and cerebellum in autism patients as shown by opposite regulation of proteins involved in myelination and synaptic regulation. These effects appeared to be associated with differential effects on glial cell function, energy metabolism and synaptic vesicle release across the two brain regions. Further molecular profiling and imaging studies on a larger brain sample set is required to increase our understanding of the molecular pathologies of autism and for validation of the current findings. Once more brain samples are available, it will be particularly important to undertake studies on a larger sample set to investigate gender and age-related changes in autism. Such more detailed and refined investigations should lead to further insights into the pathways affected in autism with the aim to increase our understanding of this debilitating disorder and facilitate the development of novel therapeutic approaches.

## Abbreviations

ADI-R: Autism Diagnostic Interview-Revised; ASD: autism spectrum disorder; BBB: blood-brain barrier; BDNF: brain-derived neurotrophic factor; CARS: Childhood Autism Rating Scale; CB: cerebellum; CKB: creatine kinase B; DSM-IV: *Diagnostic and Statistical Manual of Mental Disorders, fourth edition*; DTT: dithiotreitol; DNM2: dynamin-2; GFAP: glial fibrillary acidic protein; LC-MS^E^: liquid chromatography mass spectrometry; MAG: myelin-associated glycoprotein; MBP: myelin basic protein; MOG: myelin oligodendrocyte glycoprotein; MRI: magnetic resonance imaging; NICHD: National Institute of Child Health and Human Development; NLGN1: neuroligin; NRXL1: neurexin; PACSIN1: protein kinase C casein kinase substrate 1; PCA: principal component analysis; PFC: prefrontal cortex; PLP1: myelin proteolipid protein; PMI: post mortem interval; QC: quality control; Q-TOF: Quadrupole time-of-flight; RLN: reelin; SRM-MS: selected reaction monitoring mass spectrometry; STBXP1: syntaxin-binding protein 1; SYN2: synapsin-2; STX1A: syntaxin-1A; SYT1: synaptotagmin-1.

## Competing interests

SB is a consultant for Myriad-RBM/Psynova Neurotech Ltd. The other authors declare no competing interests.

## Authors’ contributions

JACB carried out the molecular profiling data analyses, interpreted the results, prepared the figures and tables, and wrote the manuscript. HR and PCG interpreted the results and contributing to writing and editing of the manuscript. SB conceived the study, interpreted the results and edited the manuscript. All authors read and approved the manuscript.

## Supplementary Material

Additional file 1 Demographic information for cohort of which the prefrontal cortex was analyzed. Click here for file

Additional file 2 Demographic information for cohort of which the cerebellum was analyzed. Click here for file

Additional file 3 **Principal component analysis (PCA) plots of SRM-MS data obtained from prefrontal cortex.** In the PCA plots, every run is represented as a data point and all triplicates of the same run have the same colour. Samples of both controls and patients are visualised. No segregation of samples was identified, indicating that a batch effect was not present. The accompanying text with the data points is not similar to the sample code depicted in Table 1. Click here for file

Additional file 4 **Principal component analysis (PCA) plots of SRM-MS data obtained from the cerebellum.** In the PCA plots, every run is represented as a data point and all triplicates of the same run have the same colour. Samples of both controls and patients are visualised. After removal of one outlier, the PCA plot showed no segregation of samples, indicating that a batch effect was not present. The accompanying text with the data points is not similar to the sample code depicted in Table 1. Click here for file
