# Supplementary material for: Proteomic analysis of post mortem brain tissue from autism patients: evidence for opposite changes in prefrontal cortex and cerebellum in synaptic connectivity-related proteins
Source: Mol Autism. 2014 Jul 30;5:41. doi: 10.1186/2040-2392-5-41 (PMC4131484; doi:10.1186/2040-2392-5-41)
Supplement: Additional file 3 — Principal component analysis (PCA) plots of SRM-MS data obtained from prefrontal cortex. In the PCA plots, every run is represented as a data point and all triplicates of the same run have the same colour. Samples of both controls and patients are visualised. No segregation of samples was identified, indicating that a batch effect was not present. The accompanying text with the data points is not similar to the sample code depicted in Table 1. [file 2040-2392-5-41-S3.pptx]

## Slide 1
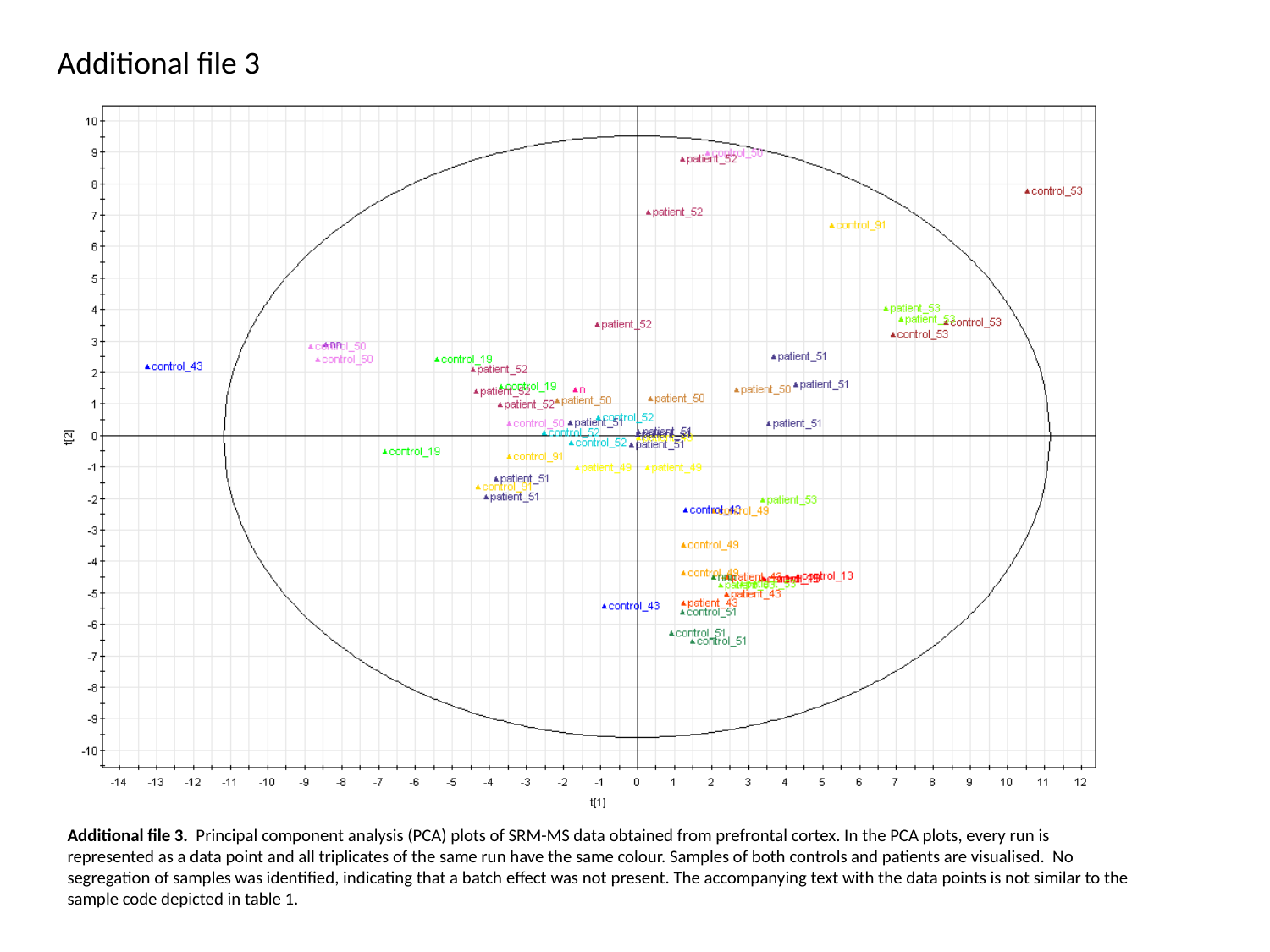

Additional file 3
Additional file 3. Principal component analysis (PCA) plots of SRM-MS data obtained from prefrontal cortex. In the PCA plots, every run is represented as a data point and all triplicates of the same run have the same colour. Samples of both controls and patients are visualised. No segregation of samples was identified, indicating that a batch effect was not present. The accompanying text with the data points is not similar to the sample code depicted in table 1.
