# Supplementary material for: Proteomic analysis of post mortem brain tissue from autism patients: evidence for opposite changes in prefrontal cortex and cerebellum in synaptic connectivity-related proteins
Source: Mol Autism. 2014 Jul 30;5:41. doi: 10.1186/2040-2392-5-41 (PMC4131484; doi:10.1186/2040-2392-5-41)
Supplement: Additional file 4 — Principal component analysis (PCA) plots of SRM-MS data obtained from the cerebellum. In the PCA plots, every run is represented as a data point and all triplicates of the same run have the same colour. Samples of both controls and patients are visualised. After removal of one outlier, the PCA plot showed no segregation of samples, indicating that a batch effect was not present. The accompanying text with the data points is not similar to the sample code depicted in Table 1. [file 2040-2392-5-41-S4.pptx]

## Slide 1
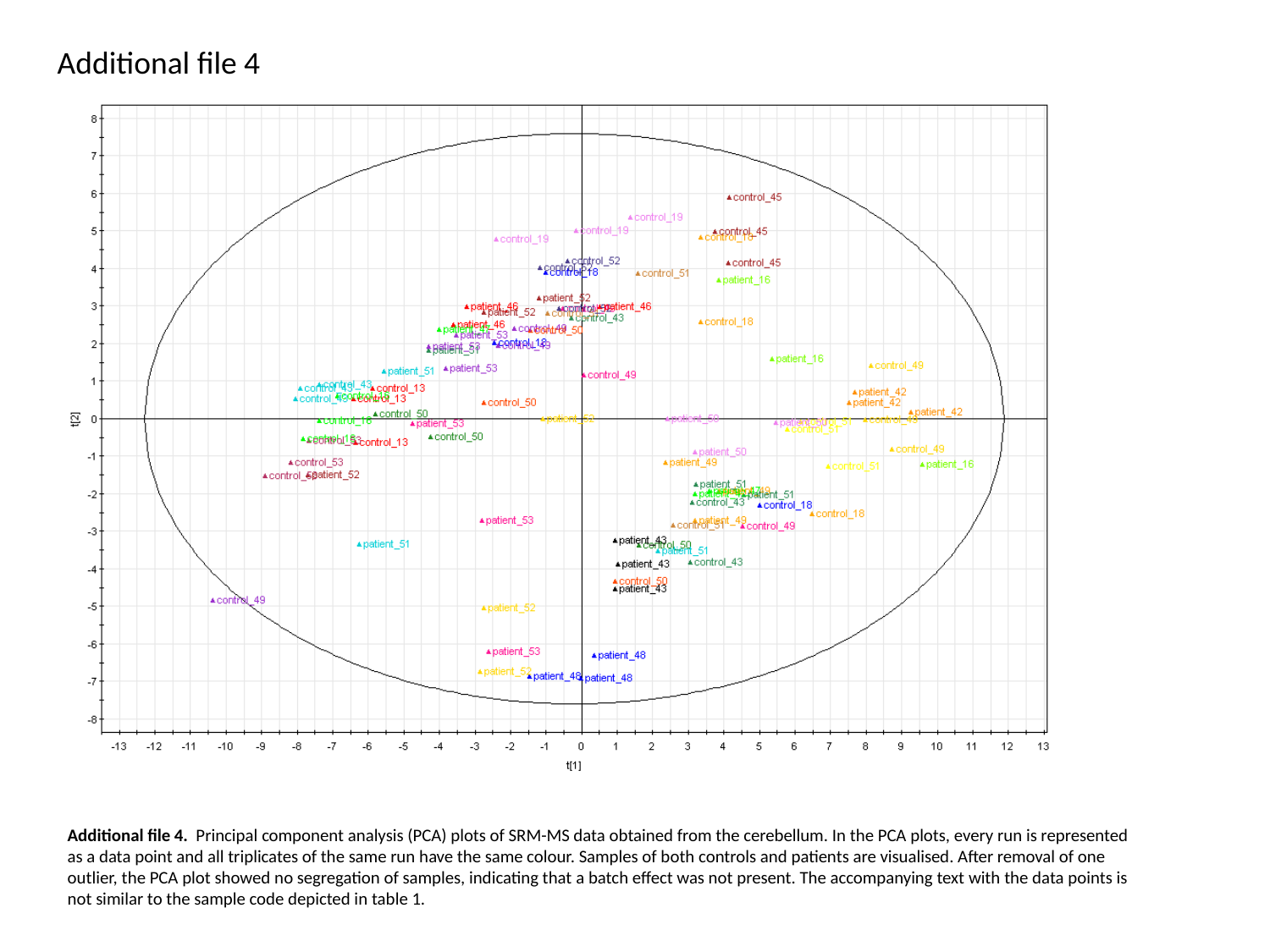

Additional file 4
Additional file 4. Principal component analysis (PCA) plots of SRM-MS data obtained from the cerebellum. In the PCA plots, every run is represented as a data point and all triplicates of the same run have the same colour. Samples of both controls and patients are visualised. After removal of one outlier, the PCA plot showed no segregation of samples, indicating that a batch effect was not present. The accompanying text with the data points is not similar to the sample code depicted in table 1.
